# Manipulation of Intestinal Antiviral Innate Immunity and Immune Evasion Strategies of Porcine Epidemic Diarrhea Virus

**DOI:** 10.1155/2019/1862531

**Published:** 2019-11-03

**Authors:** Jian Du, Junqiu Luo, Jie Yu, Xiangbing Mao, Yuheng Luo, Ping Zheng, Jun He, Bing Yu, Daiwen Chen

**Affiliations:** Institute of Animal Nutrition, Sichuan Agricultural University, and Key Laboratory of Animal Disease Resistance Nutrition Ministry of Education, Chengdu, Sichuan 611130, China

## Abstract

Porcine epidemic diarrhea virus (PEDV) infection causes watery diarrhea, dehydration, and high mortality in neonatal pigs, due to its clinical pathogenesis of the intestinal mucosal barrier dysfunction. The host's innate immune system is the first line of defence upon virus invasion of the small intestinal epithelial cells. In turn, the virus has evolved to modulate the host's innate immunity during infection, resulting in pathogen virulence, survival, and the establishment of successful infection. In this review, we gather current knowledge concerning the interplay between PEDV and components of host innate immunity, focusing on the role of cytokines and interferons in intestinal antiviral innate immunity, and the mechanisms underlying the immune evasion strategies of PEDV invasion. Finally, we provide some perspectives on the potential prevention and treatment for PEDV infection.

## 1. Introduction

Porcine epidemic diarrhea (PED) is an infectious and highly contagious viral disease in pigs characterized by anorexia, vomiting, watery diarrhea, dehydration, and body weight loss and has a high mortality, especially in neonatal pigs. The etiological agent of PED is porcine epidemic diarrhea virus (PEDV), which targets the digestive tract of pigs. Although PEDV dissemination typically occurs via the faecal-oral route, airborne transmission from the nasal cavity to intestinal mucosa is possible [1, 2].

PED was first reported in Britain, in 1971, and subsequently spread to Asian and European countries [3]. In recent years, China has experienced an increased prevalence of PEDV [4]. PEDV exists year-round, with sporadic and epidemic PED outbreak. Coinfection of PEDV with transmissible gastroenteritis virus (TGEV), porcine delta-coronavirus (PDCoV), and porcine rotavirus (PRV) is common and often causes higher mortality and morbidity in newborn piglets [5, 6]. As one of the main pathogens of diarrhea, PEDV poses a catastrophic threat to the pig industry and causes severe economic losses worldwide [7].

A wealth of information is available on the damage caused by PEDV infection to the small intestinal mucosal barrier integrity, mechanisms of inflammasome activation, and signalling cascades, but the interplay between host antiviral innate immunity and immune evasion remains unresolved. In the following sections, we will review the fundamentals of antiviral innate immunity and provide an update on recent studies regarding host antiviral innate responses. Subsequent sections depict key mechanisms that PEDV has evolved to evade virus recognition by host pattern recognition receptors (PRRs), inhibition of interferon (IFN) induction, and blocking of IFN signalling cascades. Finally, mechanisms of the host antiviral innate immune response for alleviating infection will be discussed.

## 2. PEDV

As a swine enteric coronavirus, PEDV is an enveloped, positive-sense, single-stranded RNA (ssRNA) virus [8]. The PEDV genome of 28.5 kb is polycistronic and consists of ORF1a, ORF1b, ORF3, spike protein, envelope protein, membrane protein, and nucleocapsid protein [9]. PEDV replicates in intestinal epithelial cells and alveolar macrophages of piglets via the mouth and nasal cavity, respectively, causing extensive pneumonic lesions under harsh conditions, and then invades the small intestine through the digestive tract or blood [10]. In the process of virus proliferation, PEDV damages the organelles, resulting in mitochondrial swelling and deformation, as well as dissolution of the nuclear membrane, causing cell dysfunction. Concurrently, PEDV could cause rapid destruction and detachment of the intestinal physical structure, accounting for villi atrophy or even mucosal barrier rupture in the jejunum and ileum and leading to an imbalance of the osmotic pressure, dehydration, and watery diarrhea [11]. Under the large-scale and intensive farming conditions, PED causes huge financial losses and seriously endangers the development of the pig industry.

## 3. Impairment of Intestinal Function and Barrier Integrity by PEDV Invasion

The gastrointestinal tract is not only the main site for nutrient digestion and absorption but also the target of physiological barrier function against the invasion of exogenous pathogens. Pigs exposed to high pathogen loads are stressed and unhealthy. PEDV infection in neonates markedly decreases growth performance, blocks tissue accretion, and impairs the future performance in surviving piglets [12]. Establishment of an effective invasion requires penetrating a tightly protected mucosal barrier [13]. After infected with PEDV, the intestine of piglets is filled with white-to-yellow liquid. The intestinal walls become thin and translucent, with mesentery hyperaemia [14]. The intestinal epithelial cells deteriorate, becoming flat-like immature cells. Villous structure in the small intestine becomes short and atrophied. Serious exudation and cell infiltration can be detected in the lamina propria of the mucosa [15].

The intestinal barrier function relies on the intact mechanical, chemical, biological, and immunological barriers, of which the mechanical barrier is the most important. PEDV infection significantly reduces the activities of brush border membrane-bound digestive enzymes, such as disaccharidases (lactase, sucrase, and maltase), aminopeptidase N, and alkaline phosphatase [16]. Dysfunction of the membrane ion pump and abnormal Na^+^, K^+^, Ca^2+^, and Mg^2+^ distribution eventually induce a water electrolyte imbalance characterized by diarrhea and dehydration [10]. Mucins secreted by goblet cells are essential for maintaining enteric homeostasis and intestinal epithelial integrity. During early PEDV infection, the number of goblet cells is depleted or significantly decreased in the jejunum and ileum, inducing lesions in the mucus layer, followed by infection with other enteric pathogens [17]. In addition, the expression of tight junction protein ZO-1 and adhesion junction protein E-cadherin is decreased and anomalously distributed in the small intestinal villous at 1–3 days postinoculation and reversibly recovered at 5 days postinoculation [18]. These results suggest that the integrity of the intestinal epithelial mechanical barrier is susceptible to early PEDV infection. PEDV invasion also changes the gut microbiota profiles, which may be crucial for gut health and function [19]. *Fusobacteria* and *Proteobacteria*, as pathogenic organisms, are dominant in PEDV-infected animals, while short-chain fatty acid-producing bacteria (*Prevotella*, *Bacteroides*, and *Coprococcus*) and the butyrate-producing bacterium (*Clostridium butyricum*) are dramatically decreased due to dysbiosis in the context of PEDV infection [20].

## 4. Host Innate Immune Response to PEDV Infection

In the process of virus infection and replication, the host innate immune response, as the first line of defence, can utilize PRRs to detect and respond to pathogen-associated molecular pattern (PAMP) molecules of the invading virus. PRRs mainly include retinoic acid-inducible gene (RIG) type I receptors (RLRs), toll-like receptors (TLRs), nucleotide-binding oligomerisation domain-like receptors (NLRs), and melanoma 2- (AIM2-) like receptors (ALRs) [21]. After PEDV invades host cells, its genomic nucleic acid, double-stranded RNA (dsRNA), and protein produced during replication mediate two predominant signalling pathways (the RLR signalling pathway and TLR signalling pathway) in the natural immune response to exhibit congenital antiviral function.

### 4.1. RIG-I-Like Receptor Signalling Pathway

In the resting state, the inactivated RIG-I is self-folding, and its repressor domain covers other functional domains, inhibiting caspase-recruitment domains (CARDs) from playing the role of signal transduction. When cells are infected with viruses, the C-terminal of RIG-I binds to viral RNA, resulting in conformational changes [22]. By exposing the CARD region to the outside, the downstream adapter protein mitochondrial antiviral signalling protein 5 (MAV5) is recruited. MAV5 is an IFN-*β* promoter stimulator-1 (IPS-1) with a pivotal role in the innate immune response against viral pathogens. After IPS-1 is activated, it interacts with tumour necrosis factor (TNF) receptor-associated factor 3 (TRAF3) through its proline enrichment region to recruit and activate TANK-binding kinase 1 (TBK1) and inhibitor of nuclear factor kappa B (NF-*κ*B) kinase subunit epsilon (IKK*ε*) [23]. The downstream molecules, IFN regulatory factors (IRF3 and IRF7), are phosphorylated to promote the expression of IFN-I [24]. In addition, activated IPS-1 can bind to the proline enrichment region of TRAF6/2, which activates NF-*κ*B through the IKK*α*/*β*/*γ* complex and induces the expression of inflammatory cytokines. Meanwhile, IPS-1 can also interact with Fas-associated protein with death domain (FADD), which forms a complex with caspase-8 and caspase-10 and participates in the activation of NF-*κ*B. Similarly, the protein melanoma differentiation-associated gene 5 (MDA5) can recognise viruses and relay signals to downstream critical factors IRF3, IRF7, and NF-*κ*B. MDA5 recognises dsRNA fragments larger than 1 kb, while RIG-I recognises shorter dsRNA fragments [25].

### 4.2. TLR Signalling Pathway

The TLR signalling pathway is another pathway involved in the innate immune response following PEDV invasion. Belonging to the type I transmembrane protein family, TLRs can recruit specific binding molecules, such as myeloid differentiation primary response 88 (MyD88) and toll/interleukin-1 receptor (TIR) domain-containing adapter-inducing IFN-*β* (TRIF), and initiate downstream signal transduction, leading to the production of inflammatory cytokines, chemokines, and antimicrobial peptides [26]. TLR3, TLR7, and TLR9 distribute in the cytoplasm and remain stable in the endoplasmic reticulum (ER), from which they are transported to endosomes to bind with ligands [27]. TLR3 mainly recognises the dsRNA virus genome during ssRNA virus replication. Its horseshoe-shaped structure has a large surface area to promote the recognition of viral dsRNA by viral-infected cells. The binding of dsRNA to the N- and C-termini of the outer convex surface of TLR3 facilitates the formation of homodimers through the C-terminal region. TLR3 relies on the TRIF pathway to activate IRF3 and NF-*κ*B signalling pathways and induce the expression of IFN-I and inflammatory cytokines. In plasmacytoid dendritic cells, TLR7 and TLR8 recognise viral ssRNA in the lysosome and activate NF-*κ*B and IRF7 through MyD88 to induce inflammatory cytokines and IFN-I, respectively. In addition, autophagy is involved in the transfer of ssRNA to vesicles expressing TLR7. TLR9 recognises viral and bacterial DNA. Downstream signal transduction requires the degradation of TLR9 by cytoprotease. TLR9 recruits MyD88 to activate NF-*κ*B and IRF7, leading to their phosphorylation and nuclear translocation [28]. Previous *in vitro* and *in vivo* research demonstrated that TLR2, TLR3, TLR4, TLR7, and TLR9 were involved in PEDV-induced NF-*κ*B activation in porcine intestinal epithelial cells (IECs), suggesting that the virus can exploit its surface glycoprotein and intranuclear nucleic acids to trigger the innate immunity [29, 30].

## 5. Antagonistic Strategies against Innate Immunity by PEDV

The pathogenicity of PEDV is closely related to its induced inflammatory response. Viral infection can induce innate and adaptive immune responses and produce a series of inflammatory cytokines and chemokines, which play an important role in PEDV-induced inflammatory response and resistance to PEDV evasion of the host innate immune response. Proinflammatory cytokines are important for pathogen clearance, while excessive inflammation may cause tissue damage. Cytokines and chemokines are mainly produced from three signalling pathways: NF-*κ*B, mitogen-activated protein kinase (MAPK), and IFN. Nevertheless, a great deal of evidence supports the notion that PEDV has evolved to counteract the antiviral innate immunity through manipulating the above signalling cascades.

### 5.1. NF-*κ*B Signalling Pathway

NF-*κ*B plays an important role in PEDV-induced inflammation and regulation of the immune response. Under resting conditions, NF-*κ*B is confined to the cytoplasm as inactive homodimers and heterodimers, among which p50/p65 heterodimers are most abundant, by binding with inhibitory protein I*κ*B. When the canonical pathway is triggered, I*κ*B is phosphorylated by the I*κ*B kinase (IKK) complex, which targets it for ubiquitination and proteasomal degradation, allowing NF-*κ*B to translocate to the nucleus where it can mediate the transcription of IFNs and proinflammatory cytokines [31]. In PEDV protein-expressed IECs, PEDV nucleocapsid protein and envelope protein, localised in the ER, trigger ER stress and activate NF-*κ*B, thereby resulting in the upregulation of interleukin-8 (IL-8) and B-cell lymphoma 2 (Bcl-2) [32, 33]. Coincident with the induction of the NF-*κ*B pathway in IECs, it was found that the expression of IL-1*α*, IL-1*β*, TNF-*α*, chemokine C-C ligands (CCL2 and CCL5), and C-X-C chemokine 8 (CXCL8) was increased by activating the NF-*κ*B promoter in human embryonic kidney (HEK) 293T cells transfected with the plasmid pCMV-PEDVnsp4-HA [34]. Similarly, microRNA-221-5p transfection of PEDV-infected African green monkey kidney-derived MA104 cells (MARC-145 cells) activated the NF-*κ*B signalling [35]. It is thought that the NF-*κ*B signalling is mediated through inhibiting the activities of NF-*κ*B inhibitor *α* (also known as I*κ*B*α*) and the suppressor of cytokine-signalling-1 (SOCS1) protein to promote p65 nuclear translocation [35]. However, in porcine IECs and kidney epithelial cells, PEDV nucleocapsid protein and nonstructural protein (nsp) 1 impeded the p65 translocation and prevented NF-*κ*B from binding to the positive regulatory domain (PRD) II region of the IFN-*β* promoter and, ultimately, induced transcription of type 1 IFN genes in the nucleus [36, 37]. In turn, the production of IFN-I and IFN-*λ* was antagonised. Additionally, the PEDV non-S-INDEL strain, which has more virulent pathogenicity, downregulated the NF-*κ*B signalling pathway by an inhibitory effect on TLR4, TLR7, TLR8, and TLR9, resulting in dampening the expression of cytokines in the intestinal mucosa of piglets [30]. This action might be one of the sophisticated strategies that the virus has evolved to evade the antiviral innate immunity. The discrepancy between activation and inhibition of NF-*κ*B signalling pathway could be explained by the different strains of PEDV and time-dependent infection, but further study is needed.

### 5.2. MAPK Signalling Pathway

As central regulators of the responses to various extracellular stimuli, the MAPK cascade pathways transmit signals to corresponding intracellular targets and manipulate elaborate cellular activities. The MAPK signalling pathway includes three distinct identified families: extracellular signal-regulated kinase (ERK), c-Jun amino-terminal kinase (JNK), and p38 protein kinase. ERK1/2 mainly mediates cell proliferation and differentiation. The virus-induced activation of JNK can regulate c-Jun and activator protein-1 (AP-1) transactivation function to promote the expression of proinflammatory cytokines. Similarly, multiple viruses exploit the JNK pathway to facilitate their replication or the success of viral protein expression [38]. p38 MAPK signal transduction is involved in the inflammatory response, immune response, and cell apoptosis [39]. It is demonstrated that induction of the transcription of various inflammatory factors by another enteric *alpha-coronavirus*-transmissible gastroenteritis virus reflects the outcome of simultaneous activation of AP-1 by activating JNK1/2 and NF-*κ*B [40]. PEDV can concurrently activate ERK1/2 and JNK/p38 for optimal replication; conversely, specific inhibitors of ERK1/2 and JNK/p38 significantly impair virus progeny production, viral protein expression, and RNA synthesis but are unable to restrain PEDV-mediated apoptosis [41, 42]. On the contrary, p53, a downstream protein of the JNK/p38 pathway could perpetuate IFN signalling to negatively affect PEDV replication in HEK239T cells [43]. These findings argue for the notion that PEDV, in its favour, could adjust intracellular responses including the induction of MAPK signalling pathways.

### 5.3. IFN Signalling Pathway

Although many cytokines and chemokines are produced by various host cells after virus infection, IFNs are a kind of multifunctional cytokines with the most important role in the antiviral response. Upon virus infection, the host produces IFNs quickly to antagonise the virus and establish an antiviral state in infected cells and uninfected neighbouring cells. IFNs encompass three categories: type I (*α*, *β*, *ω*, *ε*, *τ*, *κ*, *ν*), type II (*γ*), and type III IFNs (*λ*1, *λ*2, *λ*3) [44]. All IFNs can bind to their specific receptors to activate the Janus kinase (JAK) signal transducer and activator of transcription (STAT) signalling pathway to induce the expression of hundreds of IFN-stimulating genes (ISGs). A previous study suggested that the IFN system was compartmentalized, with the predominant effect of IFN-*λ* on IECs and IFN-*α*/*β* on other enteric cells [45]. Using IPEC-J2 cells, it was demonstrated that IFN-*λ* had a greater inhibition of PEDV replication than IFN-*α* and markedly upregulated the transcriptional expression of three antiviral proteins of utmost importance, namely, ISG15, myxovirus resistance A (MxA), and 2′,5′-oligoadenylate synthetase- (OAS-) directed RNaseL (OASL) [46, 47]. Although PEDV failed to infect porcine monocyte-derived dendritic cells, it could induce the transcription of IFN-I, including IFN-*α* and IFN-*β* [48].

**Figure 1:**
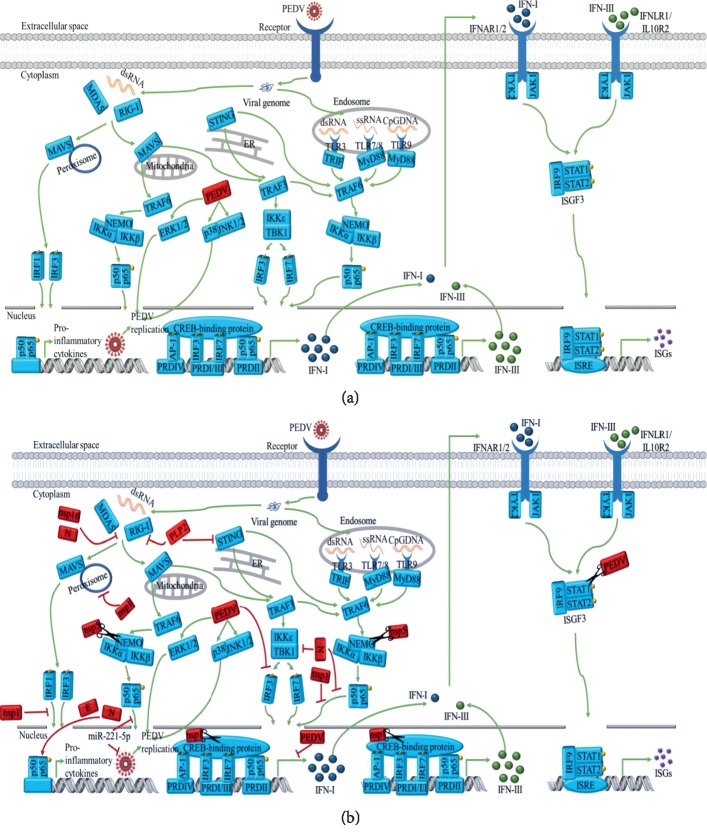
Innate immune response and the potential mechanisms of PEDV antagonising the IFN system. (a) During the cellular receptor-mediated internalisation of PEDV via direct fusion of viral-cellular membranes, its genomic nucleic acids are released into the cytosol. During replication of the virus genome, the ssRNA and dsRNA are recognised by host innate PAMPs, including endosomal TLRs (TLR3, 7/8) and cytosolic RLRs (RIG-I and MDA5). The activation of host innate PAMPs further elicit the production of proinflammatory and IFN-I and IFN-III following the nuclear translocation of transcription factors (i.e., NF-*κ*B, IRF1, IRF3, and IRF7) that bind with their respective PRD regions. Subsequently, IFN-I and IFN-III are delivered to the extracellular environment to engage with cognate receptors of both infected and noninfected neighbouring cells in autocrine and paracrine mechanisms. The activation of JAK-STAT induces the nuclear localisation of ISGF3 complex, as well as the production of hundreds of IFN-stimulating genes (ISGs) to antagonise the virus and establish an antiviral state. (b) PEDV produces viral proteins (shown in the red box) to directly antagonise the IFN system for circumvention of the host's innate immunity. Besides, PEDV can utilize ERK1/2 and JNK/p38 to promote virus replication.

Even if IFNs exert antiviral activity during viral infection, the virus could still replicate efficiently. Existing reports suggest that there are two main approaches to escape innate immunity. One of the most effective mechanisms by which enteric coronaviruses have evolved to circumvent the host's innate immunity is to directly antagonise the IFN systems, as summarized in Figure 1 [49]. Firstly, PEDV could inhibit IFN signalling transduction via regulating the activity of STAT. It has been shown that STAT1 protein degradation was blocked in PEDV-infected Vero E6 and IPEC-J2 cells treated with MG132 (a protease inhibitor), but it could not be inhibited by 3-MA (an autophagy suppressor). Meanwhile, PEDV infection enhanced the level of ubiquitinated STAT1. These observations indicate that PEDV can subvert the IFN-I response by ubiquitin-proteasome targeting degradation of STAT1 [50]. Secondly, PEDV could interrupt PRRs to avoid detection. Recent findings showed that PEDV nucleocapsid protein and nsp16 markedly inhibited the IFN-*β* promoter via subverting RIG-1/MDA5 cascades in HEK293T cells and IPEC-J2 cells [23, 51]. Subsequently, members of the antiviral IFN-induced protein with tetratricopeptide repeats (IFIT) family (IFIT1, IFIT2, and IFIT3) were inhibited [52]. Ubiquitination and deubiquitination in the cytoplasm and nucleus are critically involved in modulation of the innate immune response. Papain-like protease 2 (PLP2) as an IFN antagonist encoded by PEDV exhibited potent deubiquitinase activity to hydrolyse the cellular K48- and K63-linked polyubiquitinated chain. In that study, IFN-*β* expression was negatively regulated by deubiquitinating RIG-I and STING (STimulator of INterferon Genes) in a catalytic-dependent manner in HEK293T cells and Vero cells [53]. Thirdly, IRFs could be impeded by PEDV to prompt the antiviral state in chaos. PEDV blocked immunostimulant (polyinosinic : polycytidylic acid) mediated IRF-3 nucleus migration, possibly through attenuating the activity of PS-1 by directly targeting TBK1 to evade the innate immunity, leading to the abatement of IFN-*β* and production of ISGs in IECs [51]. IRF1 other than IRF3/7 may also play a distinctive role in mediating IFN-*λ* production in the IECs [54]. Among 21 viral proteins, 11 were found to suppress the type III IFN activities, including nsp1, nsp3, nsp5, nsp8, nsp14, nsp15, nsp16, ORF3, the envelope protein, integral membrane protein, and the nucleocapsid protein. PEDV nsp1, in particular, can potently antagonise the nuclear translocation of IRF1 by reducing the production of peroxisomes, resulting in the downregulation of IFN-*λ* production in IPEC-DQ, LLC-PK1, and MARC-145 cells [55]. Finally, owing to their various functions of the PEDV structural and nonstructural proteins, complicated relationships exist between the virus proteins and the host immune response. PEDV nsp1 can induce the proteasome-dependent degradation of the cAMP-regulated enhancer binding (CREB) protein (CBP) in the nucleus, which disrupts the enhanceosome assembly, leading to inhibition of IFN-I yield in MARC-145 cells [56]. By targeting the glutamine 231 (Q231) residue of the NF-*κ*B essential modulator (NEMO), the 3C-like protease of PEDV nsp5 could impair eventual IFN-*β* production via proteolytic cleavage of NEMO to subvert the innate immune signalling in HEK293T cells [57]. The comprehensive studies of the innate immune evasion of PEDV underscore the importance of seeking effective solutions to eliminate virus infection through the modulation of antiviral innate signalling.

## 6. Therapeutic Strategies for PEDV Infection

There is no effective treatment for the pathogen of PEDV, as yet. The PEDV vaccine cannot effectively protect swine from diarrheal infection, and the immune effect of the current PEDV vaccine still needs further improvement. Therefore, a better understanding of the rapid-responding innate immune defence mechanisms would be beneficial to improve therapeutic strategies to antagonise PEDV infection.

### 6.1. Cytokines

It is established that IFNs play a dominant role in mediating antiviral effects. Previous observations highlight the possibility that porcine IFN-*λ*3 (poIFN-*λ*3) may serve as a useful biotherapeutic candidate to inhibit PEDV infection. Furthermore, the expression of ISGs with well-known antiviral properties, including ISG15, OAS1, and Mx1, is significantly increased in Vero E6 cells [58]. In addition to the induction of antiviral mechanisms via poIFN-*λ*3, modulation of the innate immune pathway has also been attempted with recombinant mature plasmid pIL-22 (mpIL-22) possessing innate immune-modulatory activities. The antiviral activity of mpIL-22 was mediated by the STAT3 signalling pathway, through the upregulation of the antimicrobial peptide beta-defensin 2 (BD-2) and the antiviral cytokines (IL-18 and IFN-*λ*) in IPEC-J2 cells [59]. Hence, mpIL-22 could be a novel therapeutic intervention to curtail the devastating enteric diarrhea virus.

### 6.2. Amino Acids

Amino acids improve the intestinal integrity and maintain normal physiological barrier function, while amino acids preserve the local and overall immune system by modulating the expression of cytokines, thereby protecting the host from diseases [60]. The mammalian target of rapamycin (mTOR) signalling pathway plays an important role in the intestinal mucosal immune response, and thereby intestinal health. Emerging studies have shown that inhibiting the activity of mTOR could induce IEC defects and intestinal villus atrophy in mice [61]. Glutamate supplementation could increase the jejunal villus height-to-crypt depth ratio, claudin-1 protein expression, and mTOR (Ser^2448^) phosphorylation level in piglets [62]. In IPEC-J2 cells, leucine activated the mTOR signalling pathway, promoted the expression of tight junction proteins ZO-1, ZO-2, and claudin-3, and regulated protein synthesis and metabolism, including enzymatic biochemical reactions [63]. The aforementioned studies indicate that administration of amino acids can ameliorate the intestinal health status and protein synthesis via the mTOR signalling pathway. It implies that amino acids would be an influential additive to modulate the innate immunity against virus infection via mTOR signalling activation. A subsequent study demonstrated that PEDV virulent strain inhibited protein synthesis in Vero cells by downregulating mTOR activity and its downstream targets 4EBP1 and p70S6k [64]. Adding leucine in the diet improved the immune function of piglets infected with PEDV and, furthermore, alleviated the stress response of piglets [65]. *N*-acetylcysteine, an effective precursor of cysteine, decreased inflammation, enhanced the antioxidant capacity, and alleviated intestinal disruption in pigs challenged with PEDV [66]. These results substantiate that supplementation of amino acids effectively prevents pigs from PEDV infection by regulating the mTOR signalling pathway. Knockout of the tuberous sclerosis complex gene TSC2 activated mTOR, decreased the activity of NF-*κ*B, and downregulated the expression of IL-6, IL-10, and IL-12 [67]. mTOR complex 1 (mTORC1) has the ability to promote the production of type I IFN by activating IRF-5 and IRF-7. Meanwhile, IFN-I can activate phosphoinositide3-kinase- (PI3K-) Akt-mTOR signalling to regulate the expression of ISGs in certain cell types [68]. Taken together, it is speculated that amino acids can harness the innate immune antiviral activity by regulating the activity of mTOR, which plays a pivotal role in the intestinal immune response induced by PEDV.

### 6.3. Chinese Herbs

Extensive *in vivo and in vitro* experiments have proved that various Chinese herbal medicines can potently prevent viral infection. A mixture of medical herbs (*Taraxcum mongolicum*, *Viola yedoensis* Makino, *Rhizoma coptidis*, and *Radix isatidis*) attenuated the growth performance impairment and intestinal lesions of newborn piglets challenged with PEDV [69]. The combination of *Sophora flavescens* extract and stevioside could inhibit rotavirus replication and alleviate diarrhea *in vivo* [70]. Due to the abundance of biologically active compounds in herbal medicines, most of them and their secondary metabolites have been employed as potential antiviral agents.

### 6.4. Vitamins

Dietary vitamin supplementations beneficially affect animal growth, intestinal development, and immune function and can alleviate the negative effects of different stresses. Studies have shown that dietary supplementation of 200 and 500 IU vitamin D can mitigate the damage of porcine rotavirus in pigs and significantly increase the expression of RIG-I and IPS-1 and the mRNA levels of IFN-*β* and ISG15, indicating that supplementation of vitamin D activates the RIG-I signalling pathway, inducing the expression of IFNs and antiviral factors to alleviate the adverse impact of the virus [71]. Furthermore, vitamin D can block the nuclear translocation of NF-*κ*B p65, leading to the reduction of proinflammatory cytokines including IL-1*β*, IL-6, and TNF-*α*, and eventually attenuate the impairment of stress on the body [72]. The expression of proinflammatory cytokines (IL-1*β*, IL-6, TNF-*α*, and CCL20) and PRRs (TLR3, RIG-I, and MDA5) was also downregulated by 1, 25-dihydroxyvitamin D_3_ in human corneal epithelial cells [73]. These results suggest that vitamin D may suppress the downstream NF-*κ*B signalling pathway through the RLR and TLR signalling pathways to alleviate the intestinal damage caused by PEDV.

### 6.5. Others

Development of novel antiviral reagents is of utmost importance to control virus spread. Nanoparticles possessing distinctive physicochemical characteristics have shown outstanding potential advantages in antiviral activity. After treatment with silver nanoparticle-modified graphene oxide nanocomposites, PEDV nucleocapsid protein expression level markedly descended, and IFN-*α*, IFN-inducible protein 10 (IP-10), and ISGs were upregulated in MARC-145 cells [74]. Similarly, as-prepared cationic carbon dots based on curcumin-capped and glutathione-capped Ag_2_S nanoclusters markedly enhanced the production of phosphorylated IRF3 (p-IRF3) and phosphorylated p65 (p-p65) proteins, which promoted the expression of ISG20, ISG54, IL-6, and IL-8 in Vero cells. Collectively, these results could not exclude the probability that the innate immune response might be triggered *in vitro* by the cationic carbon dots and Ag_2_S nanocluster treatments [75, 76].

## 7. Conclusions

PEDV infection destroys intestinal mucosal barriers and causes intestinal injury in piglets. By regulation of RIG-1/MDA5 and TLR2/3 activities, PEDV could activate NF-*κ*B and IRF3/7 to mediate the production of inflammatory cytokines and IFNs, which subsequently activate the JAK-STAT signalling pathway to induce antiviral factors. Although multiple competent and host signalling molecules are effective in protecting the host against PEDV, these responses are still vulnerable to antagonism and subversion by pathogenic viruses. PEDV, which targets porcine enterocytes in the intestinal villi, has developed various immune evasion strategies by blocking the recognition of PRRs, suppressing the activation of NF-*κ*B and IFN production, and disrupting IFN signalling and ISGs induction. Developing effective antiviral reagents, based on the innate immunity, has shown widespread promise. Emerging studies have begun to shed light on the interference by cytokines, which directly activate the innate immune system to combat the virus. Likewise, amino acids, vitamins, and Chinese herbal medicines can be used as nutritional intervention measures, and they play an important role in regulating PEDV escape from the host innate immune response. Therefore, further investigations of the host antiviral response will benefit a deeper understanding of innate immunity for the exploration of different mitigation strategies.
